# Co-phagocytosis of VEGFA with HER2-overexpressing cancer cells induced by HER2-VEGFA–bispecific antibodies improves antitumor responses

**DOI:** 10.1172/jci.insight.194494

**Published:** 2025-09-04

**Authors:** Yang Lu, Songbo Qiu, Zhen Fan

**Affiliations:** Department of Experimental Therapeutics, The University of Texas MD Anderson Cancer Center, Houston, Texas, USA.

**Keywords:** Clinical Research, Immunology, Oncology, Therapeutics

## Abstract

We conceived of a type of antitumor mechanism of action by which a soluble target in the tumor microenvironment, such as a tumor-driving growth factor, can be phagocytized along with cancer cells via antibody-dependent cellular phagocytosis (ADCP) using an antibody bispecific for the soluble target and a solid target overexpressed on the cancer cell surface. We explored this concept through engineering bispecific antibodies (BsAbs) co-targeting human epidermal growth factor receptor-2 (HER2) and vascular endothelial growth factor A (VEGFA) in an scFv-IgG format (VHS). We showed that the HER2-VEGFA BsAbs but not the parental antibodies alone or in combination induced co-phagocytosis of VEGFA and HER2-overexpressing cancer cells by tumor-associated macrophages via ADCP. In both immunocompromised and immunocompetent mice with aggressive tumors, the BsAbs demonstrated greater anti-metastasis activity and produced a greater survival benefit than the parental antibodies alone or in combination, in a manner dependent on Fcγ receptors on the macrophages. Our results provide proof of the concept that HER2-VEGFA BsAbs achieve enhanced antitumor activity by leveraging HER2 overexpressed on the cancer cell surface to induce co-phagocytosis of VEGFA. Our findings warrant clinical testing of the strategy to treat metastasis and recurrence of HER2-overexpressing solid tumors that respond to anti-VEGFA therapy.

## Abstract 2

A novel bispecific antibody improves mouse survival by inducing co-phagocytosis of a soluble target (VEGFA) via ADCP of cancer cells overexpressing a solid target (HER2)

## Graphical abstract

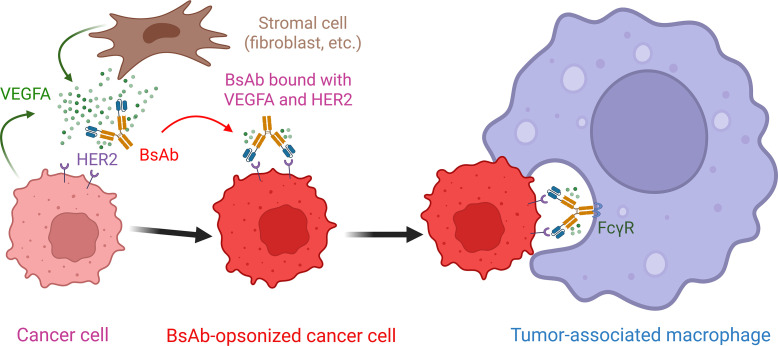

## Introduction

Bispecific antibodies (BsAbs) represent a new class of therapeutic antibodies that bind to and exert therapeutic activities against 2 different targets or 2 different epitopes on the same target (1, 2). Several bispecific targeting strategies for BsAbs have been explored (3, 4). The so-called biparatopic BsAbs bind to 2 non-overlapping epitopes on the same target, a strategy that can enhance binding avidity. Other BsAbs bind to 2 different targets that function cooperatively, complementarily, or even redundantly, such as targets involved in immune checkpoint regulation, tumor heterogeneity, or ligand redundancy, in a spatiotemporally coordinated manner and may therefore be more effective than simple combination of the 2 parental antibodies (4). Particularly compelling are BsAbs that exert new therapeutic activities through mechanisms that do not exist with simple combinations of the 2 parental antibodies (4). Such BsAbs have been called obligatory BsAbs or obligate BsAbs because BsAb binding to 2 different antigen-binding sites is obligatory for gaining a new therapeutic mechanism of action (4). A successful example of such a BsAb is blinatumomab, a CD3- and CD19-binding BsAb that bridges CD3 on T cells with CD19 overexpressed on leukemia cells and thereby acts as a T cell engager to direct the cytotoxic activity of T cells to the leukemia cells (5, 6). Another bispecific T cell engager targeting CD3 and DLL3 was granted accelerated approval by the US Food and Drug Administration (FDA) for treating DLL3-overexpressing small cell lung cancer in 2024 (7). As of March 2025, 9 BsAbs were approved by the FDA, of which 7 were approved in oncology (8). More than 50 BsAbs have been investigated in clinical trials, and more than 180 BsAbs are in preclinical development (9).

Antibody-dependent cellular phagocytosis (ADCP) is a process whereby antibody-opsonized cells, pathogens, and sometimes soluble proteins are engulfed by macrophages following the antibody’s Fc-mediated engagement of the Fc receptors on macrophages (10, 11). In this study, we conceived of a type of anti-tumor mechanism of action of a BsAb designed to have one binding site for a solid target overexpressed on the cancer cell surface, such as a receptor or marker, and one binding site for a soluble target in the tumor microenvironment (TME), such as a cytokine or other growth factor that is proven to be a key driver of tumor growth, metastasis, and/or immunosuppression. We here coined the term “co-phagocytosis” to describe the process through which soluble targets in the TME are anchored to cancer cells by the type of BsAb engineered as described, leading to internalization and degradation of the soluble targets in macrophages via BsAb-induced ADCP of co-targeted cancer cells. We hypothesized that more molecules of the soluble target would be co-phagocytized via BsAb-induced ADCP of cancer cells, because of overexpression of the solid target on the cancer cells, than via ADCP induced by an antibody specific for the soluble target only.

To validate the concept of co-phagocytosis through this bispecific targeting strategy, we selected human epidermal growth factor receptor-2 (HER2) and vascular endothelial growth factor A (VEGFA) because of the proven clinical significance of these 2 targets and an existing pipeline of respective antibody drugs already clinically approved. HER2 was initially found to be overexpressed on breast cancer cells and is now emerging as a promising target for genomically informed therapy across a variety of solid tumor types (12, 13). VEGFA is well documented as a key cancer driver that is secreted by both cancer cells and cancer stromal cells in the TME and that not only promotes tumor angiogenesis (14, 15) but also suppresses tumor immune responses (16, 17). In our studies, we generated and characterized HER2-VEGFA BsAbs to enable co-phagocytosis of VEGFA with HER2-overexpressing cancer cells through simultaneous binding to HER2 and VEGFA. We evaluated the therapeutic activities of HER2-VEGFA BsAbs against aggressive mouse tumors implanted in both immunocompromised nude mice and immunocompetent transgenic mice that are immunotolerant to HER2. In addition, we assessed the extent to which the therapeutic activity of HER2-VEGFA BsAbs depends on ADCP through co-administration of a Fcγ receptor–blocking (FcγR-blocking) antibody.

## Results

### BsAb in VHS format exhibits strong simultaneous binding to HER2 (a solid target) and VEGFA (a soluble target).

**Figure 1:**
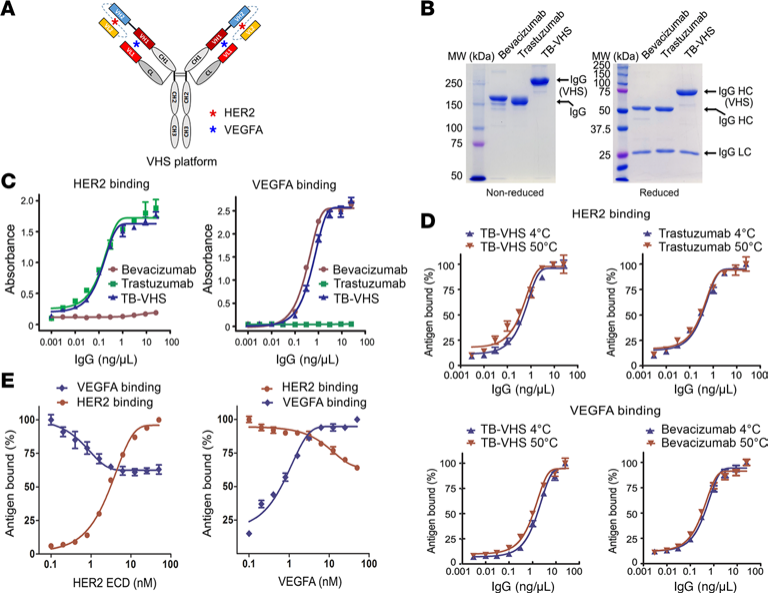
Physicochemical properties and functional characterization of TB-VHS. (**A**) Schematic illustration of the BsAb in the VHS platform. The blue asterisks represent VEGFA to which the BsAb can bind, and the red asterisks represent HER2 to which the BsAb can bind. (**B**) Coomassie blue–stained gels of TB-VHS and its parental antibodies separated by SDS-PAGE under reducing (right) and nonreducing (left) conditions. HC, heavy chain; LC, light chain; MW, molecular weight. (**C**) Specific binding of TB-VHS and its parental antibodies to HER2 and human VEGFA detected by ELISA. For detecting HER2 binding, HER2 ECD recombinant protein–coated 96-well microplates were used to capture the antibodies, and antibodies were detected by HRP-labeled anti–human IgG antibody. For detecting VEGFA binding, rabbit anti–human Fc antibody–coated 96-well microplates were used to capture the antibodies, and antibodies were incubated with biotinylated human VEGFA and detected by streptavidin-HRP conjugate. (**D**) Thermal stability of TB-VHS. TB-VHS, bevacizumab, and trastuzumab were incubated in a water bath at 50°C for 1 hour, and then binding of these antibodies and antibodies stored at 4°C against HER2 and human VEGFA was detected by ELISA as in **C**. (**E**) Competitive binding of TB-VHS to HER2 and human VEGFA. TB-VHS (5 nM) was incubated with either 5 nM biotinylated VEGFA and increasing concentrations of HER2 ECD recombinant protein (left) or 5 nM HER2 ECD recombinant protein and increasing concentrations of VEGFA (biotinylated and unlabeled) (right) in a solution at 4°C for 1 hour. Then, separate 96-well microplates coated with rabbit anti–human Fc antibody were used to capture TB-VHS. Binding of VEGFA to TB-VHS was detected by streptavidin-HRP, and binding of HER2 ECD to TB-VHS was detected by a biotinylated anti-HER2 antibody and then streptavidin-HRP.

We opted for an scFv-IgG BsAb format, in which a single chain variable fragment (scFv) derived from a parental antibody targeting a cell surface marker is fused recombinantly to the N-terminus of the heavy chain of a parental antibody targeting a soluble molecule; this platform is referred to herein as heavy chain variable domain (VH) modified with scFv (VHS) (Figure 1A). With the VHS platform, a trastuzumab-bevacizumab-VHS (TB-VHS) BsAb was engineered in a human IgG1 framework by fusing the scFv sequence of trastuzumab to the N-terminus of the VH of bevacizumab. TB-VHS had a higher molecular weight than commercial trastuzumab or bevacizumab (Figure 1B) as a result of fusion of the trastuzumab-derived scFv to bevacizumab. The binding affinity of TB-VHS for HER2 was similar to the affinity of trastuzumab for HER2, and the binding affinity of TB-VHS for human VEGFA was similar to the affinity of bevacizumab for human VEGFA (Figure 1C). Incubation of TB-VHS at 50°C for 1 hour resulted in no noticeable loss of affinity for HER2 or for VEGFA compared with the affinity of TB-VHS kept at 4°C and the respective parental antibodies kept at 4°C (Figure 1D), indicating that the protein structure of the VHS platform was stable. With the VHS platform, we detected greater than 60% of the maximal binding of TB-VHS to VEGFA in the presence of an excess of HER2 extracellular domain (ECD) recombinant protein up to 10 times the concentration of VEGFA and vice versa (Figure 1E).

**Figure 2:**
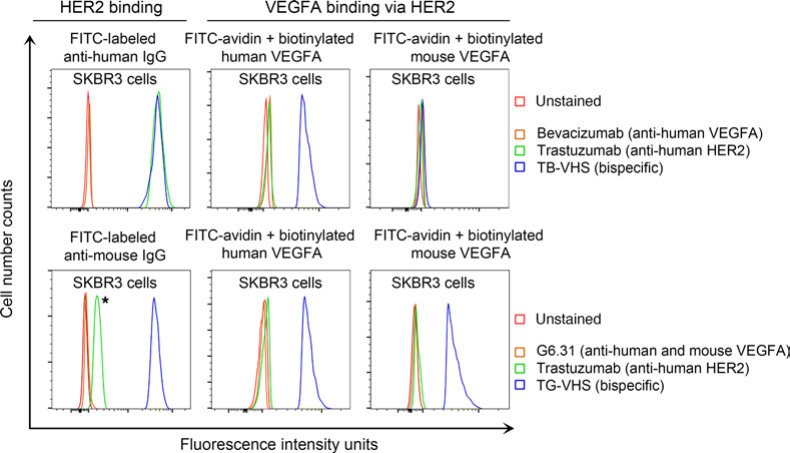
Detection of bispecific binding of TB-VHS and TG-VHS to HER2 and to human and mouse VEGFA on live cells by flow cytometry analysis. After incubation at 4°C for 1 hour with TB-VHS (upper panel) or TG-VHS (lower panel) or respective parental antibodies as shown, HER2-overexpressing SKBR3 cells were incubated with FITC-labeled anti–human IgG antibody (upper panel, left column) or FITC-labeled anti–mouse IgG antibody (lower panel, left column); FITC-avidin plus biotinylated human VEGFA (middle column); or FITC-avidin plus biotinylated mouse VEGFA (right column). The asterisk indicates minor cross-reaction of the FITC-labeled anti–mouse IgG antibody with trastuzumab (humanized IgG).

Next, we engineered a trastuzumab-G6.31-VHS (TG-VHS) BsAb by fusing the scFv sequence of trastuzumab to the N-terminus of the VH of G6.31, a monoclonal antibody that targets both human and murine VEGFA (18, 19), in a mouse IgG2a framework to permit preclinical studies in immunocompetent mice. Figure 2 shows bispecific binding of TB-VHS and TG-VHS to HER2 and human or mouse VEGFA on live cells by flow cytometry analysis. Both TB-VHS and TG-VHS bound to HER2 on SKBR3 human breast cancer cells, detected respectively by an FITC-labeled anti–human IgG antibody and an FITC-labeled anti–mouse IgG antibody (Figure 2, upper and lower left). Human VEGFA but not mouse VEGFA was detected on TB-VHS–treated SKBR3 cells (Figure 2, upper middle and right), because the anti-VEGFA component of TB-VHS is derived from bevacizumab, which binds only to human VEGFA (20, 21). By contrast, both human VEGFA and mouse VEGFA were detected on TG-VHS–treated cells (Figure 2, lower middle and right). Together, these results confirm that TB-VHS is a humanized antibody bispecific for HER2 and human VEGFA (but not mouse VEGFA), whereas TG-VHS is a mouse antibody bispecific for HER2 and human VEGFA (and mouse VEGFA as well).

### HER2-VEGFA BsAb induces co-phagocytosis of fibroblast-secreted VEGFA with HER2-overexpressing cancer cells via ADCP in co-cultures with macrophages.

To test whether VEGFA was co-phagocytized along with HER2-overexpressing cancer cells via BsAb-induced ADCP, we co-cultured 4T1 mouse mammary tumor cells lentivirally transduced to express HER2, firefly luciferase, and mCherry protein (termed 4T1-HLC cells) with RAW264.7 mouse macrophages in fresh culture medium supplemented with conditioned medium from culture of NIH3T3 fibroblasts lentivirally transduced to secrete human VEGFA fused with enhanced green fluorescent protein (EGFP) (termed NIH3T3-VG cells; “VEGFA medium”) or from culture of the fibroblasts lentivirally transduced with the EGFP construct only (termed NIH3T3-G cells; “control medium”). Flow cytometry was used to detect mCherry and EGFP fluorescence signals in the RAW264.7 macrophages following each of 5 treatments: (a) control mouse IgG; (b) G6.31.2a, constructed on the basis of G6.31 sequences but with its Fc domain re-engineered in the mouse IgG2a framework; (c) 4D5.2a, constructed on the basis of trastuzumab but with its Fc domain re-engineered in the mouse IgG2a framework; (d) G6.31.2a plus 4D5.2a; or (e) TG-VHS.

**Figure 3:**
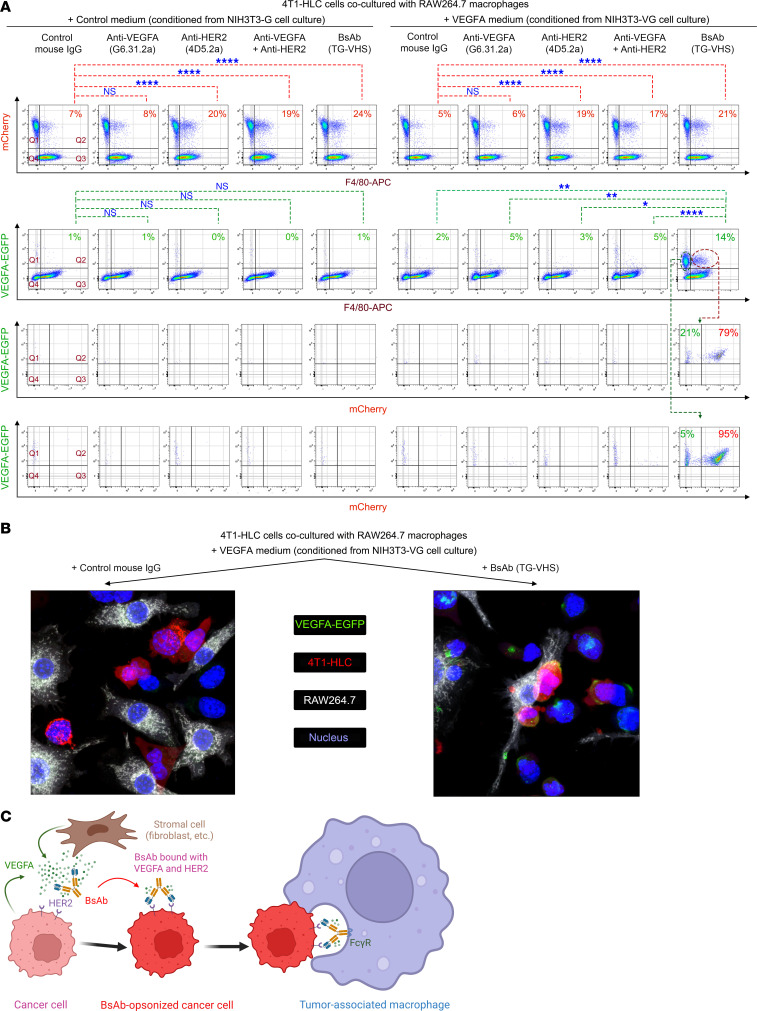
Co-phagocytosis of fibroblast-secreted VEGFA with HER2-overexpressing cancer cells via HER2-VEGFA BsAb–induced ADCP in co-cultures with macrophages. (**A**) 4T1-HLC cells and RAW264.7 macrophages (at a 1:5 cell number ratio) were co-cultured in fresh medium supplemented with conditioned medium (at a 1:1 volume ratio) from culture of NIH3T3-G cells or from culture of NIH3T3-VG cells and were treated with one of the antibodies (0.5 μg/mL each) as indicated for 2 hours in a 37°C incubator with 5% CO_2_. The cells were then harvested and stained with APC-conjugated anti-F4/80 antibody for flow cytometry analysis. Upper panel: The co-cultured cells were gated for mCherry positivity in F4/80-positive cells (indicating ADCP of HER2-overexpressing cells). The cells in Q2 indicate 4T1-HLC cells phagocytized by the macrophages in the co-culture. Phagocytosis (%) = Q2/(Q1 + Q2) × 100. Lower panel: Top row: The co-cultured cells were gated for GFP positivity in F4/80^+^ cells (indicating VEGFA co-phagocytosis via ADCP of HER2-overexpressing cells). Co-phagocytosis (%) = Q2/(Q1 + Q2) × 100. Middle row: EGFP and F4/80 double-positive cells were further gated for mCherry positivity. Bottom row: The EGFP-positive but F4/80-negative cells were further gated for mCherry positivity. NS, not significant. *P <0.05, **P < 0.01, ****P < 0.0001 by Fisher’s exact test. (**B**) RAW264.7 cells were stained with CellTrace Far Red fluorescent dye (shown in white) and then seeded on a poly-D-lysine–coated cell culture dish (FluoroDish, World Precision Instruments; https://www.wpiinc.com/var-2827-fluorodish-cell-culture-dish.html) overnight. The next day, 4T1-HLC cells (shown in red) were added to the RAW264.7 cell culture and incubated for 3 hours in the presence of TG-VHS or control antibody as indicated, along with concentrated conditioned medium from NIH3T3-VG cell culture containing VEGFA-EGFP (shown in green). The cells were counterstained with DAPI fluorescent dye (shown in blue) and then fixed with 2% paraformaldehyde prior to confocal cell imaging analysis. Original magnification, ×80. (**C**) A model depicting the process of VEGFA co-phagocytosis induced by the HER2-VEGFA BsAb. Created in BioRender (https://BioRender.com/6ibpdoc).

Figure 3A shows the basal level of phagocytosis and levels of ADCP of 4T1-HLC cells (mCherry-positive) by RAW264.7 macrophages (F4/80-APC–positive) after each of the 5 treatments in the co-cultures supplemented with control medium (left panel) or VEGFA medium (right panel). Low levels of phagocytosis were detected following control mouse IgG treatment of the co-cultures supplemented with the control medium or VEGFA medium (7% and 5%, respectively). Similar levels of phagocytosis were detected upon G6.31.2a treatment of the co-cultures supplemented with the control medium or VEGFA medium (8% and 6%, respectively). Levels of phagocytosis were significantly increased upon treatment that included a HER2-targeting antibody, including 4D5.2a (20% and 19%, respectively), G6.31.2a plus 4D5.2a (19% and 17%, respectively), and TG-VHS (24% and 21%, respectively), indicating increased levels of phagocytosis of 4T1-HLC cells by RAW264.7 macrophages via ADCP.

Figure 3A, lower panel, shows levels of co-phagocytosis of VEGFA-EGFP with 4T1-HLC cells by RAW264.7 macrophages under the same treatment conditions as in the upper panel of Figure 3A. Compared with the other treatments, TG-VHS led to a significant increase in the subpopulation of cells double positive for F4/80 and VEGFA-EGFP (Q2), from 5% or less with the other treatments to 14% with TG-VHS (Figure 3A, lower panel, right top row). Further gating of Q2 showed that 79% of the VEGFA-EGFP–positive macrophages were also positive for mCherry (Figure 3A, lower panel, right middle row), confirming that the majority of VEGFA-EGFP in the macrophages following TG-VHS treatment was co-phagocytized along with 4T1-HLC cells (mCherry-positive). Further gating of the cells positive for VEGFA-EGFP but negative for F4/80 (Q1) confirmed that 95% of these cells were also mCherry-positive (Figure 3A, lower panel, right bottom row), indicating that these cells were 4T1-HLC cells bound to VEGFA-EGFP but yet to be phagocytized by RAW264.7 macrophages.

Figure 3B shows representative confocal images of 4T1-HLC cells and RAW264.7 cells co-cultured in the presence of VEGFA medium. VEGFA-EGFP, which is a soluble protein, was undetectable in the co-culture treated with control mouse IgG (Figure 3B, left panel) but detectable in the co-culture treated with TG-VHS (Figure 3B, right panel) as a result of VEGFA-EGFP to be anchored on the 4T1-HLC cell surface and subsequently co-phagocytized along with 4T1-HLC cells by RAW264.7 cells. Together, these results support the working model depicted in Figure 3C.

### HER2-VEGFA BsAb exerts anti-metastasis activity against 4T1-HL tumors co-implanted with human VEGFA-secreting mouse fibroblasts in nude mice.

To test our working model, we examined whether the BsAb led to enhanced antitumor activity in mice with HER2-overexpressing 4T1-HL cells (similar to 4T1-HLC cells but without mCherry) and VEGFA-secreting NIH3T3-V fibroblasts (similar to NIH3T3-VG cells but secreting VEGFA rather than VEGFA-EGFP fusion protein) co-implanted in the mammary fat pad. Because HER2 and human VEGFA are immunogenic in normal mice, we performed the co-implantation experiment in nude mice. Nude mice, despite being immunodeficient, possess functional macrophages required to mediate ADCP (22). Use of nude mice also permitted use of the humanized TB-VHS BsAb we developed in this study. When the tumors were detected by IVIS imaging, on day 4 after cell implantation, treatment was initiated with control human IgG, bevacizumab, trastuzumab, trastuzumab plus bevacizumab, or TB-VHS, with antibodies administered twice a week in line with practices commonly reported in the literature (23–26). To achieve a molar mass of TB-VHS equal to that of the parental antibodies at 100 μg/mouse when used alone and at 2 × 100 μg/mouse when used in combination, we opted for 150 μg of BsAb per mouse to account for the higher molecular weight (~225 kDa) of TB-VHS than of the parental antibodies (~150 kDa each), owing to the scFv fusion (Figure 1, A and B).

**Figure 4:**
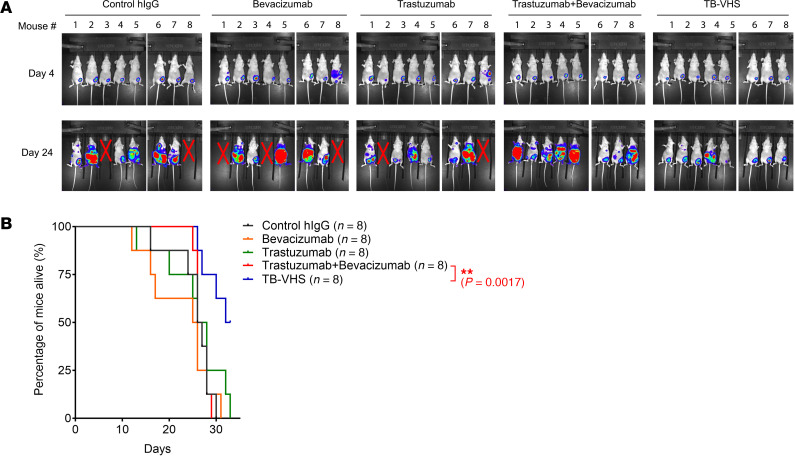
Anti-metastasis activity of TB-VHS against 4T1-HL tumors co-implanted with human VEGFA-secreting mouse fibroblasts in nude mice. (**A**) 4T1-HL mouse mammary tumor cells were co-implanted with NIH3T3 fibroblasts transduced to express human VEGFA in the mammary fat pad of nude mice. The mice were divided into 5 groups with 8 mice per group. Starting on day 4, mice were injected intraperitoneally twice a week with control human IgG (hIgG) (100 μg/mouse), bevacizumab (100 μg/mouse), trastuzumab (100 μg/mouse), trastuzumab plus bevacizumab (100 μg/mouse + 100 μg/mouse), or TB-VHS (150 μg/mouse, equivalent to 100 μg in molar mass of conventional antibody). The mice were imaged using IVIS on day 4 and day 24. X indicates mice that died before day 24. (**B**) Survival curves of the mouse treatment groups described in **A**. **P < 0.01 by log-rank test.

Tumor metastasis was detected on day 10, and shortly after, the mice started to die of massive metastasis. A simple combination of trastuzumab and bevacizumab produced no advantage over either single treatment alone in controlling the extent of metastasis; however, TB-VHS–treated mice had remarkably less extensive metastasis than mice in the other groups had on day 24 (Figure 4A). The mice in all groups were treated as scheduled until all mice in the control group had died and the number of mice remaining in any of the treatment groups became too small to permit statistical comparisons. On day 34, when all mice in the other groups had died, 4 of the 8 mice treated with TB-VHS remained alive (Figure 4B). However, tumors persisted in the surviving mice and, owing to the aggressiveness of the tumor model, the mice eventually died. Nevertheless, the results showed greater antitumor activity of TB-VHS than of the parental antibodies used alone or in combination.

### HER2-VEGFA BsAb induces co-phagocytosis of recombinant VEGFA with HER2-overexpressing cancer cells via ADCP in co-cultures with primary bone marrow–derived macrophages.

**Figure 5:**
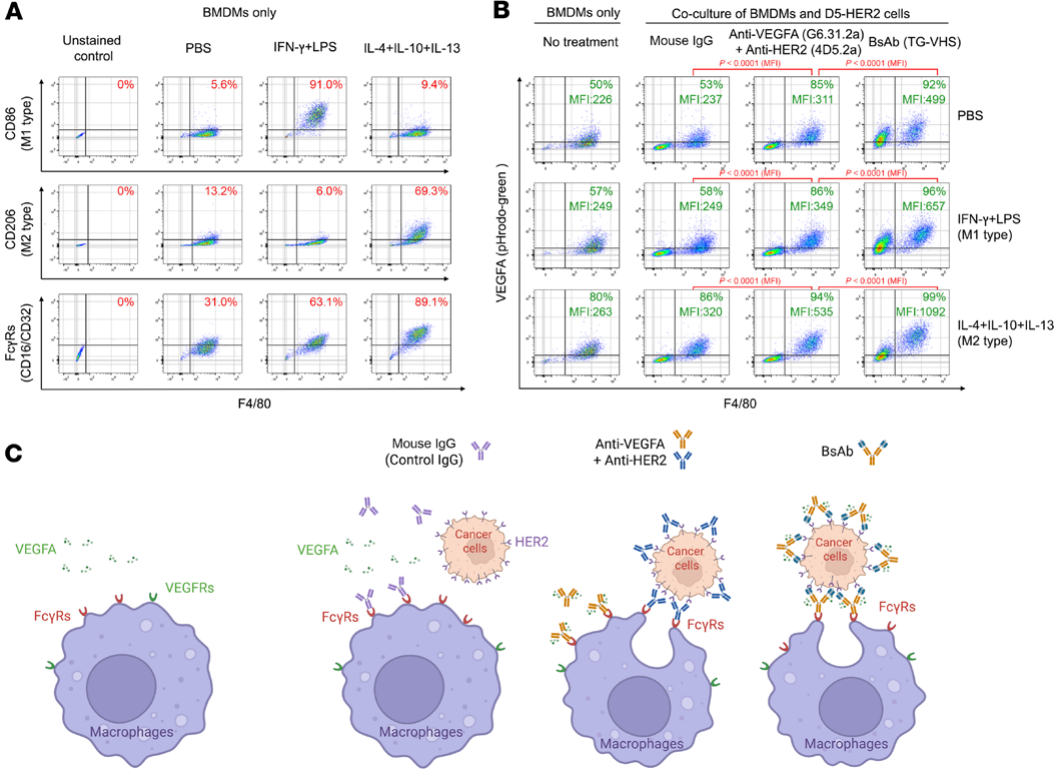
Co-phagocytosis of recombinant VEGFA with HER2-overexpressing cancer cells via HER2-VEGFA BsAb–induced ADCP in co-culture with unpolarized and polarized BMDMs. (**A**) BMDMs were treated with IFN-γ (50 ng/mL) and LPS (1:1000 dilution of the stock) for 12 hours or with IL-4, IL-10, and IL-13 (50 ng/mL each) for 24 hours in culture as indicated. The BMDMs were then subjected to FcγR blockade with anti-CD16/CD32 antibody and staining with APC-conjugated anti-F4/80 antibody, PE-conjugated anti-CD86 antibody, and PE/Cy7-conjugated anti-CD206 antibody, or subjected to FcγR blockade with PE-conjugated anti-CD16/CD32 antibody and staining with APC-conjugated anti-F4/80 antibody. The flow cytometry data were gated using FlowJo software (v10) for the levels of CD86, CD206, and FcγRs on F4/80-positive cells. The gating strategy for flow cytometry analysis is shown in Supplemental Figure 1; supplemental material available online with this article; https://doi.org/10.1172/jci.insight.194494DS1 (**B**) BMDMs unpolarized or polarized as shown in **A** were mixed with D5-HER2 cells (at a 1:2 ratio of cell number) in the presence of mouse IgG control antibody, combination of G6.31.2a and 4D5.2a, or TG-VHS (0.5 μg/mL each). The cell mixture or the BMDMs alone were incubated with a pHrodo green–labeled recombinant VEGFA (0.5 μg/sample) for overnight. The cell samples were then analyzed by flow cytometry after staining with APC-conjugated anti-F4/80 antibody. The flow cytometry data were gated using FlowJo software (v10) for VEGFA green positivity in F4/80-positive cells (BMDMs) and F4/80-negative cells (D5-HER2). The gating strategy for flow cytometry analysis is shown in Supplemental Figure 2. *P* < 0.0001 by Wilcoxon’s test. (**C**) Models depicting the processes of phagocytosis and co-phagocytosis of VEGFA opsonized upon the treatments. Created in BioRender (https://BioRender.com/wwe8c21).

To further validate our working model, we harvested primary bone marrow cells from normal mice and differentiated them into bone marrow–derived macrophages (BMDMs) by culture of the cells with macrophage colony–stimulating factor (M-CSF) for 7 days. The BMDMs were left unpolarized or further polarized to M1-type macrophages by treatment with a combination of interferon γ (IFN-γ) and lipopolysaccharide (LPS) or to M2-type macrophages by treatment with a combination of interleukin 4 (IL-4), IL-10, and IL-13. Flow cytometry analysis of the BMDMs showed a marked increase in the level of CD86 (a marker of M1-type macrophages) after treatment with IFN-γ and LPS and CD206 (a marker of M2-type macrophages) after treatment with IL-4, IL-10, and IL-13, confirming successful polarization of the BMDMs (Figure 5A). Flow cytometry analysis also showed that the level of FcγRs detected by 2.4G2 antibody, which recognizes both FcγRIII/CD16 and FcγRII/CD32 (27, 28), was higher in the polarized BMDMs than in the unpolarized cells.

These unpolarized or M1- or M2-polarized BMDMs were then co-cultured with syngeneic D5 mouse melanoma cells transduced to overexpress HER2 (termed D5-HER2 cells) for further characterization of co-phagocytosis of a recombinant VEGFA (labeled with pH-sensitive pHrodo green) after overnight treatment in the presence of control mouse IgG, a combination of anti-VEGFA (G6.31.2a) and anti-HER2 (4D5.2a) antibodies, or TG-VHS. Whereas the green fluorescence from VEGFA-EGFP fusion protein in Figure 3 was detectable by flow cytometry upon anchorage of VEGFA-EGFP on the cell surface with or without subsequent internalization, the green fluorescence from recombinant VEGFA labeled with pH-sensitive pHrodo green was detectable only after the VEGFA was internalized and transported to the endosomes or was phagocytized and transported to the phagosomes in the treated cells. As shown in the top row in Figure 5B, in BMDMs (F4/80-positive) cultured alone, a basal level of green fluorescence was detected, suggesting the presence of VEGFA receptors on the surface of the BMDMs. In the BMDMs co-cultured with D5-HER2 cells (F4/80-negative) and treated with the combination of the anti-VEGFA and anti-HER2 antibodies, the level of green fluorescence was higher, suggesting that additional VEGFA molecules opsonized by the anti-VEGFA antibody were endocytosed and transported to the phagosomes in the BMDMs. In the BMDMs co-cultured with D5-HER2 cells and treated with TG-VHS, the level of green fluorescence was higher than the level after treatment with the parental antibody combination, and importantly, only in TG-VHS–treated co-culture was the green fluorescence detected in both the D5-HER2 cells and the BMDMs. In the co-cultures of polarized BMDMs with D5-HER2 cells, findings followed the same pattern, but levels of green fluorescence were higher, particularly with the M2-type BMDMs (Figure 5B, middle and bottom rows). These findings are consistent with the observation of increased levels of FcγRs, which are key mediators of ADCP induction (29, 30), in the M1- and M2-polarized BMDMs (Figure 5A).

Together, these results further validate the working model illustrated in Figure 5C, in which TG-VHS opsonizes VEGFA and induces VEGFA co-phagocytosis by leveraging HER2 overexpressed on the cancer cell surface via ADCP, which can be mediated by both unpolarized and M1- or M2-polarized BMDMs, a proposed mechanism of VEGFA co-phagocytosis that is not achievable with the parental antibodies alone or in simple combination.

### HER2-VEGFA BsAb improves tumor-free survival in immunocompetent hmHER2Tg mice with aggressive D5-HER2 tumors.

**Figure 6:**
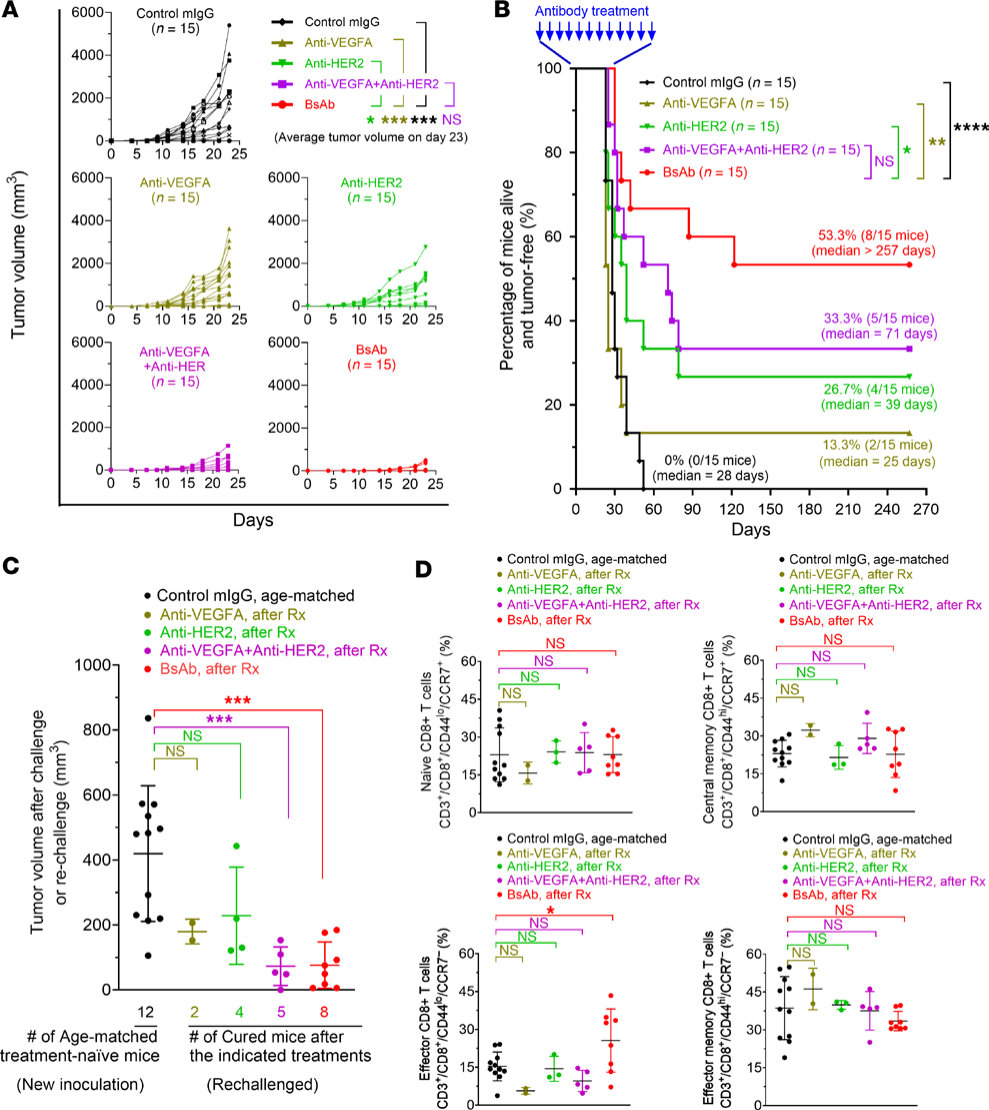
Improvement of tumor-free survival of hmHER2Tg mice with D5-HER2 tumors by TG-VHS compared with combination of anti-VEGFA and anti-HER2 antibodies. (**A**) Tumor growth in individual mice in each treatment group. D5-HER2 syngeneic tumor cells (2.5 × 10^5^ cells/mouse) were implanted subcutaneously into hmHER2Tg mice. The next day (day 1), the mice were randomly divided into 5 groups, and treatment was initiated. Mice received a control mouse IgG (mIgG) (100 μg/mouse), G6.31.2a anti-VEGFA antibody (100 μg/mouse), 4D5.2a anti-HER2 antibody (100 μg/mouse), G6.31.2a plus 4D5.2a (100 μg/mouse + 100 μg/mouse), or TG-VHS (150 μg/mouse, equivalent to 100 μg in molar mass of conventional antibody) intraperitoneally twice per week for 6 weeks. Tumor measurement was stopped after day 23, when mice started to die or had to be euthanized owing to moribund status. (**B**) Survival curves of the mouse treatment groups described in **A**. Treatments were stopped after 6 weeks, and the mice were monitored for survival up to 257 days. (**C**) Tumor challenge and rechallenge in hmHER2Tg mice. Age-matched treatment-naive hmHER2Tg mice were challenged, and the mice in **A** and **B** that remained tumor-free during the extended period (257 days) were rechallenged with equal amounts (2.5 × 10^5^ cells/mouse) of the same parental D5 tumor cells (without HER2 overexpression). Tumor growth was measured twice per week for 2 weeks. Plots show tumor volume in individual mice on day 14. (**D**) Immunophenotypic analysis of the spleen specimens from the mice in each group in **C**. Single-cell suspensions were prepared for flow cytometry analysis for the relevant markers shown. *P < 0.05; **P < 0.01; ***P < 0.001; ****P < 0.0001 by unpaired *t* test (**A**), log-rank test (**B**), or 1-way ANOVA with Dunnett’s multiple-comparison test (**C** and **D**). Rx, treatment; NS, not significant.

**Figure 7:**
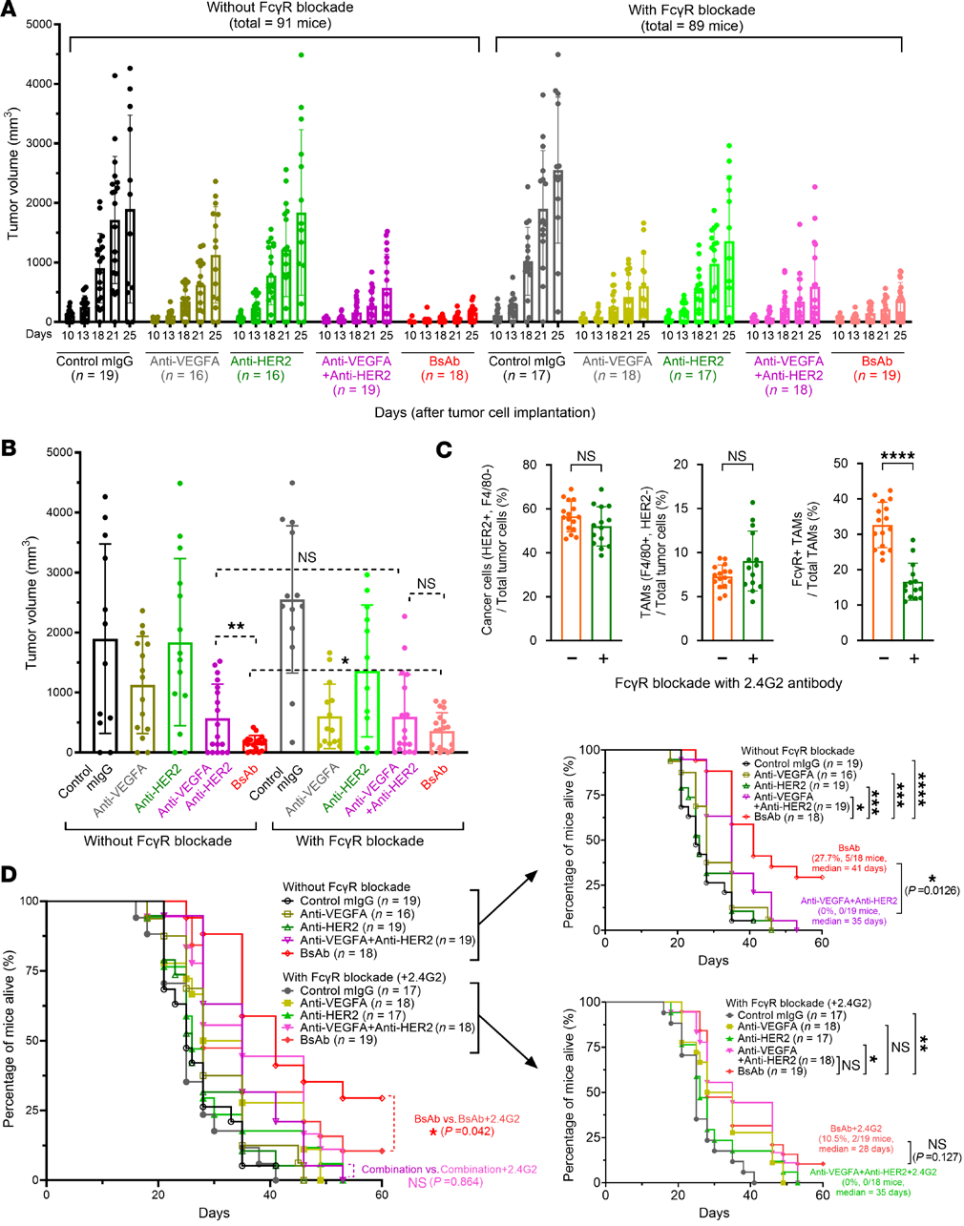
Dependence on FcγRs on TAMs of TG-VHS’s antitumor activity and survival benefit in hmHER2Tg mice with D5-HER2 tumors. (**A**) Tumor growth in individual mice after the indicated treatments without and with FcγR blockade. D5-HER2 syngeneic tumor cells (2.5 × 10^5^ cells/mouse) were transplanted subcutaneously into hmHER2Tg mice. The mice were then randomly divided into 2 cohorts receiving 2.4G2 FcγR-blocking antibody (100 μg/mouse) or not on the same day. On day 3, the mice in each cohort were further randomly divided into 5 groups, and treatment was initiated. Mice received a control mouse IgG (mIgG) (100 μg/mouse), G6.31.2a anti-VEGFA antibody (100 μg/mouse), 4D5.2a anti-HER2 antibody (100 μg/mouse), G6.31.2a plus 4D5.2a (100 μg/mouse + 100 μg/mouse), or TG-VHS (150 μg/mouse, equivalent to 100 μg in molar mass of conventional antibody) intraperitoneally twice per week for 5 weeks. Tumor measurement was stopped after day 25, when mice started to die or had to be euthanized owing to moribund status. (**B**) Statistical comparison of tumor growth on day 25 between the major groups of interest. (**C**) Analysis of results of pharmacological blockade of FcγR ex vivo. Shown are flow cytometry data from tumor samples collected from moribund mice that were euthanized on day 33. The samples within each cohort were grouped, and the grouped samples were analyzed by flow cytometry after staining with a mixture of antibodies, including BV421-conjugated anti-HER2 antibody, APC-conjugated anti-F4/80 antibody, and PE-conjugated nonspecific goat anti–rabbit IgG antibody. (**D**) Survival curves of all the mouse treatment groups described in **A** and **B** (left panel) and of the mouse treatment groups without (right panel, upper) and with (right panel, lower) FcγR blockade. *P < 0.05; **P < 0.01; ***P < 0.001; ****P < 0.0001 by unpaired *t* test (**B** and **C**) or log-rank test (**D**). NS, not significant.

To further investigate the extent to which the proposed mechanism of action by the BsAb leads to enhanced antitumor activity, we implanted D5-HER2 syngeneic melanoma cells subcutaneously in hmHER2Tg mice, a transgenic mouse line in the C57BL/6 background that is immunocompetent but immunotolerant to HER2 (31–33). D5-HER2 cells implanted subcutaneously in hmHER2Tg mice grow very aggressively and can kill the mice (similar to the killing of mice by 4T1-HL cells in Figure 4). In experiments similar to the one described in Figure 4 but with mouse antibodies used instead, the hmHER2Tg mice were randomly divided into 5 groups for treatment with control mouse IgG, G6.31.2a anti-VEGFA antibody, 4D5.2a anti-HER2 antibody, G6.31.2a plus 4D5.2a, or TG-VHS twice per week for 6 weeks, with treatment started on the day after D5-HER2 cell implantation (day 1) (Figure 6). Figure 6A shows tumor development in each mouse in each treatment group (*n* = 15 in each group) from the day of tumor cell implantation (day 0). Tumor measurement was stopped after day 23, when the mice in the control group started dying. Figure 6B shows tumor-free survival in the different groups. By day 52, all 15 mice treated with the control mouse IgG had died (median survival, 28 days). On day 257, approximately four and a half months after no further deaths occurred in the treatment groups, 2 of 15 mice (13.3%) treated with G6.31.2a (median survival, 25 days), 4 of 15 mice (26.7%) treated with 4D5.2a (median survival, 39 days), and 5 of 15 mice (33.3%) treated with G6.31.2a plus 4D5.2a (median survival, 71 days) were alive and tumor free. By contrast, 8 of 15 mice (53.3%) treated with TG-VHS were alive and tumor free (median survival, >257 days). However, despite the clear survival benefit of TG-VHS treatment, the difference in survival between the TG-VHS group and the G6.31.2a plus 4D5.2a group was not statistically significant, which prompted us to conduct another experiment with a redesigned treatment plan (shown below in Figure 7).

The mice that were alive and tumor free at the end of the experiment (day 257) were considered cured and were rechallenged with equal amounts of parental D5 melanoma cells without HER2. Age-matched treatment-naive mice were inoculated with equal amounts of D5 melanoma cells and were used as controls. Compared with the growth of D5 tumor implants in the control mice, the growth of D5 tumor implants in the cured mice treated with an anti-VEGFA activity (G6.31.2a alone, G6.31.2a plus 4D5.2a, or TG-VHS) was remarkably slower (Figure 6C). The difference was statistically significant between the control mice and the TG-VHS and combination treatment groups but not the G6.31.2a-alone group, owing to the low number of mice that survived. All mice were euthanized 2 weeks after the inoculation or rechallenge for collecting the spleens for CD8^+^ T cell immunophenotype analysis. No significant differences were found in the subpopulations of naive CD8^+^ T cells (CD3^+^CD8^+^CD44^lo^CCR7^+^), central memory CD8^+^ T cells (CD3^+^CD8^+^CD44^hi^CCR7^+^), or effector memory CD8^+^ T cells (CD3^+^CD8^+^CD44^hi^CCR7^–^); however, the subpopulation of effector CD8^+^ T cells (CD3^+^CD8^+^CD44^lo^CCR7^–^) was significantly more abundant in the mice treated with TG-VHS than in the mice in the other groups (Figure 6D), indicating that effector CD8^+^ T cells might have been activated in response to TG-VHS therapy.

### HER2-VEGFA BsAb’s antitumor activity and survival benefit depend on FcγRs on macrophages.

To confirm the role of VEGFA co-phagocytosis via BsAb-induced ADCP (Figures 3 and 5) in improving antitumor responses to the BsAbs leading to a survival benefit (Figures 4 and 6), we examined the extent to which inhibition of ADCP affected tumor responses to the antibody treatments. Specifically, we treated the mice with and without co-administration of the 2.4G2 antibody to pharmacologically block FcγRs on tumor-associated macrophages (TAMs) in vivo. Following implantation of D5-HER2 cells in hmHER2Tg mice, the mice were randomly assigned to 2 large cohorts, one receiving 2.4G2 antibody starting on day 0 (the day of tumor cell implantation) and the other not receiving 2.4G2 antibody. BsAb and other antibody treatments were started on day 3, when tumors were well established (in contrast with the experiment in Figure 6, in which treatment was started on day 1). Mice in both large cohorts were randomly divided into 5 groups (*n* = 16 to 19 per group) for treatment with control mouse IgG, G6.31.2a, 4D5.2a, G6.31.2a plus 4D5.2a, or TG-VHS twice per week for 5 weeks (in contrast with the 6 weeks of treatment for the experiment in Figure 6). In both cohorts, starting on day 18, mice in various treatment groups died or had to be euthanized owing to being moribund (data not shown). Measurement of tumor growth was stopped after day 25, when more than 50% of the mice in the control antibody groups had died, leaving too few mice for detection of statistically significant effects. However, in both cohorts, all remaining mice in all treatment groups were continuously treated as scheduled until their death or completion of the planned treatment. Figure 7A shows tumor volume in each mouse in each treatment group from day 10 to day 25 after tumor cell implantation in mice without FcγR blockade (left panel) and with FcγR blockade (right panel). In both cohorts, TG-VHS produced the best tumor growth inhibition, and the combination treatment was better than 4D5.2a or G6.31.2a alone. Figure 7B shows tumor volume in each mouse in each treatment group on day 25. In the mice without FcγR blockade, tumor growth inhibition was significantly greater for TG-VHS treatment than for the combination treatment, whereas in the mice with FcγR blockade, the difference between TG-VHS and the combination treatment was notably smaller and not statistically significant.

To confirm that 2.4G2 antibody treatment blocked the FcγRs on TAMs in the mice as expected, we analyzed tumor samples from mice euthanized on day 33, when a relatively large number of mice (*n* = 30) happened to be identified as moribund and euthanized. Sixteen tumor samples from mice without FcγR blockade (1 treated with control IgG, 6 with G6.31.2a alone, 3 with 4D5.2a alone, 5 with the combination, and 1 with TG-VHS) were combined into one group, and 14 tumor samples from mice with FcγR blockade (2 treated with G6.31.2a alone, 2 with 4D5.2a alone, 4 with the combination, and 6 with TG-VHS) were combined into another group. We analyzed the percentage of TAMs that had FcγR remaining available (FcγR^+^ TAMs) for ex vivo interaction with the Fc region of a nonspecific goat anti–rabbit IgG conjugated with phycoerythrin (PE) fluorescent dye through detection of the fluorescence by flow cytometry. As shown in Figure 7C, cancer cells (HER2-positive and F4/80-negative) and TAMs (F4/80-positive and HER2-negative) represented 56.6% ± 6.7% and 7.3% ± 1.3%, respectively, of the total tumor cells harvested from the tumors in the cohort without 2.4G2 antibody treatment, and 52.0% ± 8.6% and 9.0% ± 3.3%, respectively, of the total tumor cells harvested from the tumors in the cohort with 2.4G2 treatment. The differences between the 2 cohorts were not statistically significant. However, the percentage of TAMs with FcγR remaining available (FcγR^+^ TAMs/total TAMs) for ex vivo interaction with the PE-conjugated, nonspecific goat anti–rabbit IgG was significantly lower in the tumor samples from the cohort with 2.4G2 antibody treatment (16.6% ± 5.0%) than in the tumor samples from the cohort without 2.4G2 antibody treatment (32.7% ± 6.2%). This finding corroborates that co-administration of 2.4G2 antibody blocked FcγRs on the TAMs in the mice and thereby diminished the access of the treatment antibodies to the FcγRs.

Owing to the aggressiveness of the tumor model, all mice treated with control IgG died by day 40 (Figure 7D). On day 60, when all mice in the other treatment groups had died, 5 of the 18 mice (27.7%) in the TG-VHS group without FcγR blockade and 2 of the 19 mice (10.5%) in the TG-VHS group with FcγR blockade remained alive. Importantly, the median survival time for the mice treated with TG-VHS was 41 days without FcγR blockade compared with 28 days with FcγR blockade. This statistically significant 13-day improvement in median survival represents a 46% (41 days/28 days) improvement in median survival time. By contrast, for the other treatments, the median survival times were similar in the groups without and within FcγR blockade: 25 days and 25 days, respectively, for control mouse IgG; 28 days and 31.5 days, respectively, for G6.31.2a; 26 days and 26 days, respectively, for 4D5.2a; and 35 days and 35 days, respectively, for the combination treatment. The fact that reduced median survival time in the mice with FcγR blockade was observed only for TG-VHS treatment supports our proposed working model of a role of VEGFA co-phagocytosis induced by the HER2-VEGFA BsAb via ADCP of HER2-overexpressing cancer cells in leading to enhanced antitumor activity (Figure 3C and Figure 5C).

## Discussion

In this work, we proposed a bispecific targeting strategy and assessed this strategy by developing and testing a pair of BsAbs designed to induce co-phagocytosis of VEGFA (an example of a soluble target in the TME) via inducing ADCP of cancer cells overexpressing HER2 (an example of a solid target overexpressed on cancer cells). Our preclinical assessment of the strategy showed that our HER2-VEGFA BsAbs exerted greater anti-metastasis activity against aggressive tumor models, leading to a greater survival benefit, than did the parental anti-HER2 and anti-VEGFA antibodies used alone or in simple combination.

Depending on the desired functions or purposes, BsAbs can be engineered to be symmetrical or asymmetrical in molecular structures and can vary in molecular size from small scFv-based structures consisting of merely the variable regions of the parental antibodies linked by a flexible peptide to large, complex IgG-like molecules retaining the IgG Fc domain of the parental antibodies (1, 3, 4, 34). Our VHS BsAb platform used in the work reported here was designed to include the IgG Fc domain, not only to engage with FcγRs on macrophages for induction of ADCP but also to retain the pharmacokinetically favorable characteristics of antibody-based therapeutics (35, 36). Simultaneous bispecific binding to a soluble target in the TME and a solid target on the cancer cell surface is critical for our proposed mechanism of action; however, some BsAb platforms, despite their strong binding to 2 different targets individually, have a limited capacity to bind to the 2 different targets simultaneously. This can be the case when the respective binding sites on the hypervariable complementarity-determining regions within the variable regions of the BsAb are in proximity, leading to intramolecular steric hindrance between the 2 targets (37). Within our VHS platform, we observed remarkably less competition between HER2 and VEGFA for simultaneous binding to the BsAb than was previously reported for a “2-in-1” BsAb design (37). One potential disadvantage of the VHS platform, however, is its relatively large size as a result of scFv fusion, which may theoretically impact the penetration of the BsAb into solid tumors. Nevertheless, the size does not appear to be a major concern given that our BsAbs induced strong tumor responses in our in vivo studies. Future studies will be needed to further characterize the pharmacokinetic properties and biodistribution of the BsAbs with reference to the properties of their respective parental antibodies.

Using a cell co-culture system, we found that VEGFA-EGFP signal was detected both in cancer cells (4T1-HLC cells) and in macrophages (RAW264.7) upon TG-VHS treatment, which supports our proposed working model (Figure 3C) in which VEGFA co-phagocytosis upon TG-VHS treatment occurs via a 2-step process. In step 1, soluble VEGFA in the medium is anchored onto cancer cells following bispecific binding of TG-VHS to VEGFA and HER2. In step 2, the anchored VEGFA is phagocytized along with the targeted cancer cells by RAW264.7 macrophages via ADCP induced by TG-VHS. When we expanded the antibody treatment to overnight and used primary BMDMs, which exhibited a higher phagocytic activity than established RAW264.7 macrophages, we further confirmed a higher level of VEGFA uptake by the BMDMs in co-culture with cancer cells after treatment with the BsAb than after treatment with the combination of anti-VEGFA and anti-HER2 antibodies. Interestingly, we also found that the polarized BMDMs (M1 type or M2 type) mediated a higher level of TG-VHS–induced VEGFA co-phagocytosis than did the unpolarized BMDMs, which are considered resting macrophages in an inactive state or M0 type. This finding is consistent with the observation of higher levels of FcγRs in the polarized macrophages than in the unpolarized macrophages. This finding is interesting because TAMs are often polarized, most often to the M2 type. Of note, although the 2.4G2 antibody can recognize both FcγRIII/CD16 and FcγRII/CD32 (27, 28), an increase in FcγRs in polarized BMDMs was reported to be mainly due to an increase in FcγRII/CD32 (38).

In our studies, we developed 2 HER2-VEGFA BsAbs: TB-VHS, a fully humanized IgG1 antibody, and TG-VHS, a murine IgG2a antibody counterpart that functions analogously to TB-VHS in terms of the IgG effector functions in the species of the host (mouse) (39). The target specificity of their respective parental antibodies, trastuzumab and bevacizumab, is well documented as both are FDA-approved, and the target specificity of G6.31 was well characterized in literature (18, 19). Our findings from the nude mice with 4T1-HL tumors provided evidence that TB-VHS worked better than trastuzumab and bevacizumab alone or in combination through mechanism(s) without participation of the host immune system. We performed 2 experiments with our BsAb in immunocompetent hmHER2Tg mice with D5-HER2 tumors: one in which we started treatment on day 1 after tumor cell inoculation (Figure 6) and the other in which we started treatment on day 3 after tumor cell inoculation, when tumors were well established (Figure 7). For all of the treatments except the control mouse IgG control, overall survival at the end of the experiment was lower in the mice with treatment started on day 3 (Figure 7) than in the mice with treatment started on day 1 (Figure 6). Owing to this lower overall survival, the difference in overall survival between the TG-VHS–treated mice without and with FcγR blockade seemed small in the group with treatment started on day 3. Nevertheless, in this experiment, the median survival time, which is often used in clinical trials to evaluate the effectiveness of a new treatment, was shortened from 41 days in the mice treated with BsAb alone to 28 days in the mice treated with BsAb plus FcR blockade. This statistically significant 13-day difference represents a 46% difference in median survival time and translates to a difference of approximately 533 days in humans, as 1 mouse day is equivalent to approximately 41 human days (40). For the other treatments, the median survival times were not shortened with the addition of FcR blockade. This finding supports a functional role of FcγR in mediating enhanced activity of TG-VHS but not the other treatments. We acknowledge that the 2.4G2 antibody might also block CD16 on NK cells, leading to inhibition of antibody-dependent cell-mediated cytotoxicity (i.e., ADCC) or affect the complement system, leading to inhibition of antibody-mediated complement-dependent cytotoxicity (i.e., CDC), either of which would affect tumor response not only to TG-VHS but also to 4D5.2a alone or in combination with G6.31.2a. However, we found no significant difference in the antitumor activity of 4D5.2a alone or G6.31.2a plus 4D5.2a between the cohorts receiving and not receiving 2.4G2 antibody. Together, the findings support the conclusion that VEGFA co-phagocytosis via ADCP is a key mechanism by which TG-VHS exerts strong antitumor activity. The strong antitumor activity may be due to depletion of VEGFA in the TME upon BsAb-induced VEGFA co-phagocytosis with targeted cancer cells by the macrophages, compared with mainly sequestration of VEGFA by the action of the parental VEGFA antibodies. This mechanism by the BsAbs may be particularly helpful to treat HER2-overexpressing tumors resistant to standard anti-HER2 therapies but still sensitive to anti-VEGFA therapy.

VEGFA is a key modulator of host innate immune responses with suppressive effects in addition to its proangiogenic activities (16, 17). Several mechanisms have been reported by which VEGFA suppresses tumor immune responses, including promoting M2 polarization of macrophages and thereby causing them to secrete antiinflammatory cytokines, such as IL-10 and TGF-β (41); inhibiting dendritic cell maturation (42); upregulating immune checkpoint regulators, such as indoleamine 2,3-dioxygenase (42) and PD-L1/2 in myeloid cells (42, 43); and recruiting myeloid-derived suppressor cells and regulatory T cells into tumors (44, 45). Anti-VEGFA therapy can improve host adaptive tumor immune responses via vascular normalization, leading to increased tumor-infiltrating lymphocytes, such as CD4^+^ and CD8^+^ T cells (43, 46). We found that the BMDMs in our studies expressed VEGFA receptors, consistent with findings in the literature (41, 47). Strong inhibition of VEGFA by TG-VHS may have modulated these VEGFA’s immunosuppressive activities and thereby promoted an antitumor immune response in the hmHER2Tg mice. It is of interest that mice that were inoculated with D5-HER2 cells and became tumor free after TG-VHS treatment rejected rechallenge with the parental D5 melanoma cells (without HER2). However, D5 melanoma cells express a low level of MHC-I (H-2K^b^ and H-2D^b^), and D5-HER2 cells express an even lower level of MHC-I than parental D5 cells do (31, 32). This finding, therefore, should be interpreted with caution regarding whether the presence of effector CD8^+^ T cells contributed to tumor-free survival of the mice after TG-VHS treatment. Nevertheless, the finding that the effector CD8^+^ T cell population was increased upon TG-VHS treatment confirms that inhibition of VEGFA can enhance host immune responses by modulating immune cells, including T cell function.

Last, VEGFA signaling is important for maintaining normal function and homeostasis in vital organs, so anti-VEGFA therapy can cause toxic effects (48–50). In our experiments, we found that mice well tolerated all antibody treatments, including the BsAb treatment, which was given twice per week for 5 to 6 weeks and was stopped when the mice in the control group started to die from tumor burden and metastasis. However, the toxicity of anti-VEGFA therapy is known to depend on treatment dose and duration (48–50), so caution is warranted in the design of further mechanistic and preclinical and clinical studies of HER2-VEGFA BsAbs.

In summary, our findings support the concept of a proposed mechanism of action exerted by HER2-VEGFA BsAbs in the VHS platform that leads to a survival benefit. A limitation of current study is that only syngenetic tumor models were investigated to permit assessment of the bispecific strategy targeting both tumor and host-derived VEGFA in an immunocompetent host. A future direction of this line research will be to test the strategy in humanized mouse models with patient-derived xenografts. Given that the BsAbs improved the median survival in mice with aggressive murine tumors that are lethal due to massive metastases, it is reasonable to expect that the humanized BsAb (TB-VHS) will produce similar outcomes against patient-derived xenografts. Such expected results will warrant clinical testing of the BsAb strategy for patients with metastatic HER2-overexpressing cancers across a variety of cancer types, in particular cancers that respond to bevacizumab, such as colorectal cancer. Furthermore, our proof of the concept of the proposed mechanism of action exerted by HER2-VEGFA BsAbs suggests that the VHS platform could be applied to develop additional BsAbs with a target overexpressed on the cancer cell surface (e.g., EGFR, CD19, or CD20) and a soluble target abundant in the TME (e.g., IL-10 or TGF-β) to achieve desired activities against various types of tumors.

## Methods

### Sex as a biological variable.

Both male and female mice were included in this study, and findings were similar in the 2 sexes.

### Animals.

Female Swiss nude mice were ordered from a mouse colony facility maintained by the Department of Experimental Radiation Oncology, The University of Texas MD Anderson Cancer Center. The original transgenic breeding pairs of hmHER2Tg mice (C57BL/6 background) were provided by Louis M. Weiner (Georgetown University Medical Center, Washington, DC, USA) (31, 32). The hmHER2Tg mice have been maintained at MD Anderson Cancer Center by Zhen Fan’s team since then (33). Both male and female hmHER2Tg mice were used in this study.

### Mouse tumor models.

Two mouse tumor models were used in this study. In the first model, 4T1-HL tumor cells and NIH3T3-V cells were generated via lentiviral transduction of the respective parental cells with the respective cDNA construct(s) of interest (HER2 and firefly luciferase for 4T1-HL, VEGFA for NIH3T3-V) packed using the pLEX lentivirus packing system. 4T1-HL cells (1 × 10^5^ cells) were implanted along with NIH3T3-V cells (5 × 10^5^ cells) in the mammary fat pad of female Swiss nude mice (4–6 weeks old). In the second model, D5 melanoma cells and D5 melanoma cells expressing HER2 (D5-HER2) were provided by Louis M. Weiner (31, 32). D5 cells and D5-HER2 cells (each at 2.5 × 10^5^ cells/mouse) were implanted subcutaneously in male and female hmHER2Tg mice (8–12 weeks old).

### Mouse treatments and analysis of tumor development and mouse survival in response to the treatments.

TB-VHS, TG-VHS, 4D5.2a, and G6.31.2a antibodies were produced in-house as described below under *Engineering and production of BsAbs with the VHS platform and respective parental antibodies*. Trastuzumab and bevacizumab were purchased from the pharmacy at MD Anderson Cancer Center. Non–pharmaceutical-grade 2.4G2 anti-mouse CD16/CD32 antibody (clone 2.4G2) was purchased from Selleckchem.

Female nude mice were used to assess the therapeutic activity of TB-VHS and respective parental antibodies; details are given in the legend for Figure 4. Tumor development and tumor response to various treatments were monitored weekly in live mice by IVIS imaging. Both male and female hmHER2Tg mice were used to assess the therapeutic activity of TG-VHS and respective parental antibodies; details are given in the legend for Figure 6. Tumor volumes were measured twice per week with calipers and calculated using the formula volume = π/6 × *ab*^2^ (*a*, length; *b*, width; *a* > *b*). For both nude mice and hmHER2Tg mice, animals were euthanized when they became moribund or when their tumor size reached greater than 2.0 cm in diameter.

### Tumor rechallenge experiment.

Age-matched treatment-naive hmHER2Tg mice and the hmHER2Tg mice cured after various treatments in the experiment underwent subcutaneous implantation of equal numbers of parental D5 melanoma cells (2.5 × 10^5^ cells/mouse); this was followed by close observation of tumor development and tumor size measurement in the mice as described above.

### Immunophenotypic analysis of mouse spleen specimens.

Mouse spleen specimens were minced mechanically and then passed through a 70-μm mesh cell strainer to isolate single cells. Single-cell suspensions (0.5 × 10^6^ to 1 × 10^6^ cells/sample) were first subjected to FcγR blockade with TruStain FcX antibody (anti-mouse CD16/CD32, clone 93, BioLegend) and co-stained with Ghost Dye Violet 510 (Tonbo Biosciences), and then were stained with respective fluorescently conjugated antibodies (1–2 μL in 100 μL of flow cytometry staining buffer [0.5% BSA in PBS]). The fluorescently conjugated primary antibodies included PE-conjugated anti–mouse CD8a (clone 53-6.7), violetFluor 450–conjugated anti–human/mouse CD44 (clone IM7), PE-Cy7–conjugated anti–mouse CD3e (clone 145-2C11), APC-conjugated anti-F4/80 (clone RM8), and PerCP/Cy5.5-conjugated anti-CCR7 (clone 4B12), which were purchased from Tonbo Biosciences or BioLegend. After staining for 30 minutes at 4°C in the dark, the cells were washed twice with cytometry staining buffer and then analyzed by using a Becton Dickinson flow cytometer. The flow cytometry data were analyzed by using FlowJo software (v10) (https://flowjo.com/flowjo10/overview) to select subpopulations of cells gated successively on the fluorescence of interest.

### Analysis of pharmacological blockade of FcγR ex vivo.

Tumor specimens collected from the cohorts of mice treated with or without blockade of FcγR (2.4G2 antibody, 100 μg/mouse) were processed for single-cell suspension and then stained for flow cytometry analysis as described above in *Immunophenotypic analysis of mouse spleen specimens* with fluorescently conjugated primary antibodies, including BV421-conjugated anti-HER2 (clone 24D2, BioLegend), APC-conjugated anti-F4/80 (clone BM8, BioLegend), and PE-conjugated nonspecific goat anti–rabbit IgG (catalog sc-3739, Santa Cruz Biotechnology).

### Harvest of BMDMs from mice.

Primary BMDMs were harvested from normal mice following protocols in the literature (51, 52).

### Cell culture.

The cell lines used in the study were obtained from the American Type Culture Collection unless otherwise stated. All the cell lines except primary BMDMs were cultured in DMEM/F12 medium (50:50, v/v) supplemented with 10% FBS, 2 mM glutamine, 100 U/mL penicillin, and 100 μg/mL streptomycin. BMDMs were cultured in RPMI 1640 medium supplemented with 50 ng/mL M-CSF (SinoBiological), 10% FBS, 2 mM glutamine, 100 U/mL penicillin, and 100 μg/mL streptomycin. All cells were incubated at 37°C with 5% CO_2_ in a humidified atmosphere.

### Polarization of BMDMs in culture.

For inducing M1-type polarization, BMDMs were treated with a combination of IFN-γ (50 ng/mL; SinoBiological) and LPS (1:1000 from a 500× stock; eBioscience) for 12 hours in culture. For inducing M2-type polarization, BMDMs were treated with a combination of IL-4, IL-10, and IL-13 (SinoBiological) at 50 ng/mL of each for 24 hours in culture. The outcome of polarization was confirmed by flow cytometry analysis as described above in *Immunophenotypic analysis of mouse spleen specimens* after FcγR blockade and staining with anti-CD86 (clone GL-1, BioLegend) for M1-type macrophages or anti-CD206 (clone C068C2, BioLegend) for M2-type macrophages.

### Analysis of BsAb-induced co-phagocytosis of VEGFA via ADCP of HER2-overexpressing cancer cells in co-culture with macrophages.

For induction of co-phagocytosis of VEGFA-EGFP by TG-VHS in co-culture of 4T1-HLC cells with RAW264.7 mouse macrophages (at a 1:5 ratio of cell number), equal volumes of conditioned medium collected from culture of NIH3T3-VG fibroblasts or from culture of NIH3T3-G fibroblasts were added respectively to the co-culture of 4T1-HLC and RAW264.7 cells on a 6-well culture plate supplemented with fresh medium. The co-cultured cells were then treated with control mouse IgG, G6.31.2a, 4D5.2a, G6.31.2a plus 4D5.2a, or TG-VHS for 2 hours in a 37°C incubator with 5% CO_2_. The culture medium was then removed, and the cells were washed and harvested. After FcγR blockade and staining with APC-conjugated anti-F4/80 antibody, the cell samples were analyzed by flow cytometry as described above in *Immunophenotypic analysis of mouse spleen specimens*.

For induction of co-phagocytosis of pHrodo green–labeled recombinant VEGFA by TG-VHS in co-culture of cancer cells with primary BMDMs, recombinant VEGFA (50 μg; SinoBiological) was labeled with pHrodo green fluorescent dye (100 μg) according to the protocol of the pHrodo iFL microscale labeling kit provided by the vendor (Invitrogen). The labeled VEGFA (0.5 μg/10 μL) was added to BMDMs (2 × 10^5^ cells) in 500 μL of medium cultured without or with D5-HER2 cells (4 × 10^5^ cells, i.e., at a 1:2 ratio of cell number) in the presence of mouse IgG, G6.31.2a plus 4D5.2a, or TG-VHS (at 5 μg/mL each) in a FACS tube in a 37°C incubator overnight. The cell samples were then subjected to FcγR blockade and staining with APC-conjugated anti-F4/80 antibody for flow cytometry analysis as described above in *Immunophenotypic analysis of mouse spleen specimens*.

### Laser scanning confocal microscopy.

Laser scanning confocal microscopy was performed on a Zeiss LSM 880 using the Airyscan detector and 40× 1.2 N.A. water Apochromat objective with a 2× zoom. Images were captured as *Z*-stacks with an interval of 1 μm, range of 12 μm, and pixel size of 80 nm. Airyscan processing was performed in Zen Black (Zeiss) using default strength parameters. *Z*-stacks were processed in Zen Blue (Zeiss) image analysis software to generate maximum intensity projections as indicated. Experimental and control samples were imaged and processed in parallel using the same acquisition parameters (e.g., transmission, gain, etc.) and processing parameters.

### Engineering and production of BsAbs with the VHS platform and respective parental antibodies.

The complementarity-determining region sequences used for construction of TB-VHS, TG-VHS, 4D5.2a, and G6.31.2a expression vectors were obtained from the public domain and are published (53). The VH and VL sequences of 4D5.2a were based on those of trastuzumab, and the VH and VL sequences of G6.31.2a were based on those of G6.31, but the Fc domain of 4D5.2a and G6.31.2a was engineered in a mouse IgG2a framework to ensure comparability with the Fc domain of TG-VHS for IgG effector functions. Codon-optimized DNA fragments encoding the antibody genes were synthesized, constructed, subcloned into a homemade construct, and expressed in CHO-DG44 cells, which were cultured in chemically defined serum-free medium. The antibodies produced by the CHO-DG44 cells were then purified by subjecting the conditioned medium to binding to protein A–ceramic HyperD F affinity chromatography sorbent (Pall Laboratory), followed by acid elution with 0.1 M glycine (pH 2.6) and immediate neutralization with 1 M Tris-HCl (pH 11). Samples of protein A–purified antibodies were quantified using the Pierce Coomassie Plus (Bradford) colorimetric protein assay (Thermo Fisher Scientific) and were separated by SDS-PAGE, followed by gel staining with Coomassie blue and then soaking in a destaining solution overnight to clean background. The purified antibodies were dialyzed with PBS, then sterilized via filtration through a 0.2-μm membrane device, and kept at –80°C in aliquots before use.

### Characterization of BsAbs for binding to HER2 and VEGFA individually and competitively.

Binding of purified TB-VHS to its respective targets (HER2 and VEGFA) was measured in a cell-free PBS solution by ELISA. For detecting HER2 binding, 96-well microplates coated with HER2 ECD recombinant protein (SinoBiological) were used to capture TB-VHS or the parental anti-HER2 antibody. The captured antibodies were then detected by HRP-labeled anti–human IgG antibody (catalog 109-035-127, Jackson ImmunoResearch) in an ELISA. For detecting VEGFA binding, 96-well microplates coated with goat anti–human or anti–mouse IgG Fc antibody (catalog 109-005-098 and 115-005-062, Jackson ImmunoResearch) were used to capture TB-VHS or the parental anti-VEGFA antibody. The captured antibodies were then incubated with biotinylated human or mouse VEGFA (G&P Biosciences) and detected by streptavidin-HRP (Invitrogen) in an ELISA.

For assessing competitive binding of the 2 targets to TB-VHS, either TB-VHS (5 nM) was incubated in a solution with biotinylated VEGFA at a fixed concentration (5 nM) and HER2 ECD recombinant protein at increasing concentrations at 4°C for 1 hour, or TB-VHS (5 nM) was incubated in a solution with HER2 ECD recombinant protein at a fixed concentration (5 nM) and biotinylated VEGFA (mixtures with unlabeled VEGFA) at increasing concentrations at 4°C for 1 hour. The reaction products were then applied to separate 96-well microplates coated with anti–human IgG Fc antibody (to capture TB-VHS). VEGFA bound to TB-VHS was detected by streptavidin-HRP in an ELISA, whereas HER2 ECD bound to TB-VHS was detected by streptavidin-HRP after incubation with a biotinylated anti-HER2 antibody (catalog AHO0919, Invitrogen).

The capacity of TB-VHS or TG-VHS for binding to VEGFA following binding of the BsAbs to HER2 overexpressed on live cells was measured by flow cytometry. After incubation at 4°C for 1 hour with TB-VHS or TG-VHS or with the respective parental antibodies, HER2-overexpressing SKBR3 human breast cancer cells were incubated with FITC-labeled goat anti–human IgG antibody or FITC-labeled anti–mouse IgG antibody (catalog 109-095-098 and 115-095-003, Jackson ImmunoResearch); FITC-avidin (R&D Systems) plus biotinylated human VEGFA; or FITC-avidin plus biotinylated mouse VEGFA. The cells were then analyzed by flow cytometry as described above in *Immunophenotypic analysis of mouse spleen specimens*.

### Statistics.

Statistical analyses were performed with GraphPad Prism 10 software. The log-rank test was used to compare the survival distributions of 2 or more groups of mice after various treatments. Experimental data in the animal studies are presented as mean ± SEM. A 2-tailed, unpaired Student’s *t* test was used to compare tumor volumes between 2 groups in mice and the ex vivo and in vitro samples after various treatments. Fisher’s exact test was used to compare the percentages of mCherry-positive (or EGFP-positive) or mCherry-negative (or EGFP-negative) cells in macrophages (F4/80-positive cells) of flow cytometry data. Wilcoxon’s test was used to compare the median fluorescence intensity value of flow cytometry data between 2 groups. Dunnett’s 1-way ANOVA was used to compare the means of multiple data groups to a control group. In all cases, a *P* value of less than 0.05 was considered significant.

### Study approval.

All mouse experiments were performed in accordance with guidelines and protocols approved by the Institutional Animal Care and Use Committee of MD Anderson Cancer Center, Houston, Texas, USA.

### Data availability.

Values for all data points in graphs and analysis are reported in the Supporting Data Values file. Materials can be obtained upon reasonable request by contacting the corresponding author.

## Author contributions

ZF conceived the original idea, acquired the funding, supervised the project, and wrote the manuscript. YL, SQ, and ZF designed the study. YL and SQ performed the experiments. YL, SQ, and ZF analyzed the data. All authors approved the final version of the manuscript. YL was given first listing as YL initiated the project.

## Funding support

• NIH grant R01CA262288.

• Breast Cancer Research Foundation grants BCRF-18-051, 19-051, 20-051, 21-051, 22-051, 23-051, and 24-051.

## Supplementary Material

Supplemental data

Unedited blot and gel images

Supporting data values

## Version 1 09/04/2025

In-Press Preview

## Version 2 10/22/2025

Electronic publication
