# Supplementary material for: Co-phagocytosis of VEGFA with HER2-overexpressing cancer cells induced by HER2-VEGFA–bispecific antibodies improves antitumor responses
Source: JCI Insight. 2025 Sep 4;10(20):e194494. doi: 10.1172/jci.insight.194494 (PMC12581669; doi:10.1172/jci.insight.194494)
Supplement: Supplemental data [file jciinsight-10-194494-s176.pdf]

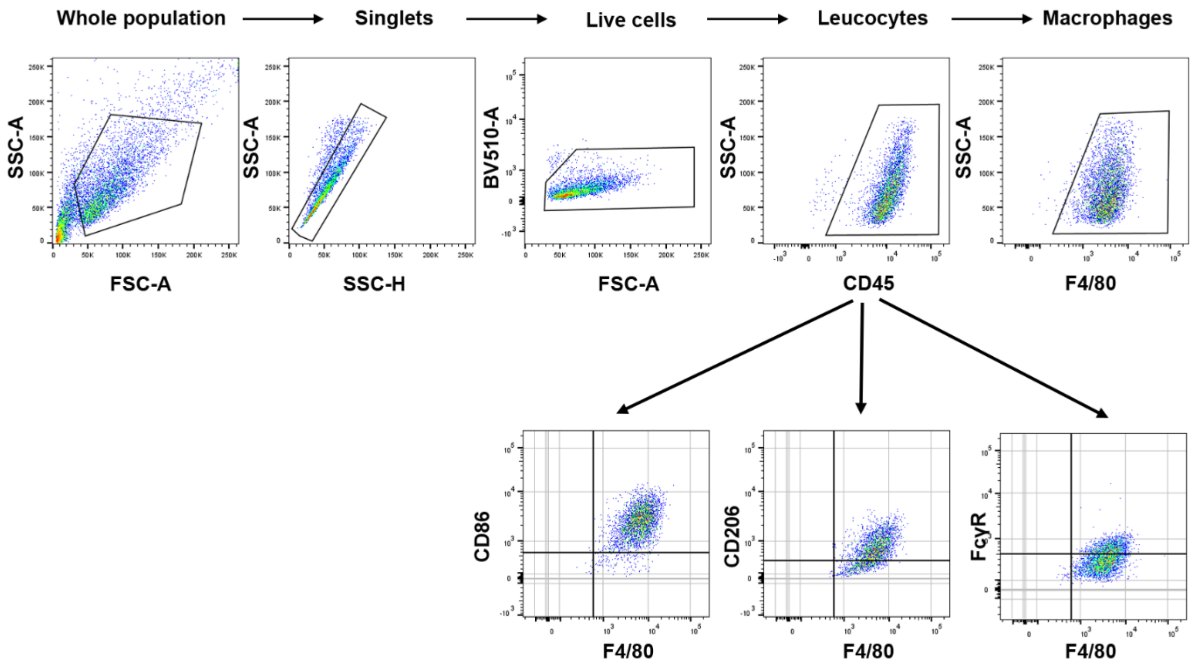


**Supplemental Figure 1. Gating strategy for the flow cytometry experiment in Figure 5A.** Shown is the gating strategy used for analysis of the BMDMs in F4/80 versus SSC-A dot plot and in F4/80 versus CD86, F4/80 versus CD206, and F4/80 versus FcγR bivariate dot plots.

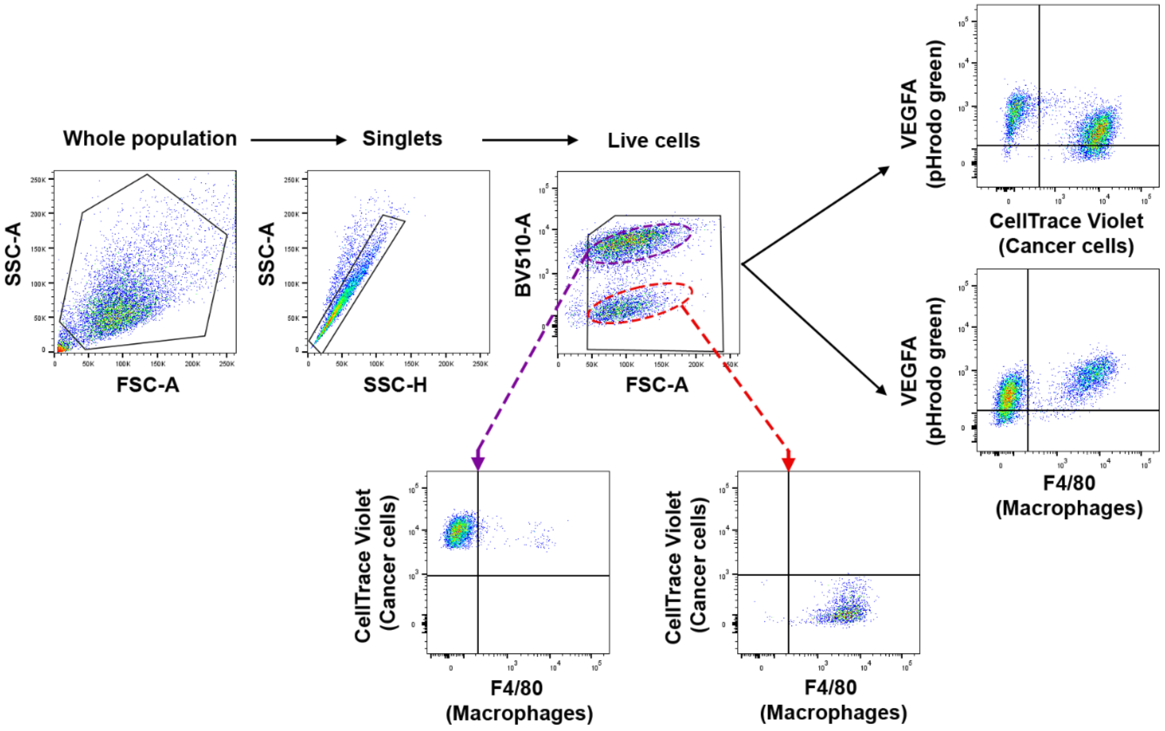


**Supplemental Figure 2. Gating strategy for the flow cytometry experiment in Figure 5B.**

Shown is the gating strategy used for analysis of the co-cultured cancer cells (stained with CellTrace Violet) and BMDMs (F4/80 positive) in CellTrace Violet versus VEGFA, F4/80 versus VEGFA, and F4/80 versus CellTrace Violet bivariate dot plots.
