# Supplementary figures and images for: Co-phagocytosis of VEGFA with HER2-overexpressing cancer cells induced by HER2-VEGFA–bispecific antibodies improves antitumor responses
Source: JCI Insight. 2025 Sep 4;10(20):e194494. doi: 10.1172/jci.insight.194494 (PMC12581669; doi:10.1172/jci.insight.194494)

# Unedited blot and gel images

Figure 1B, part 1

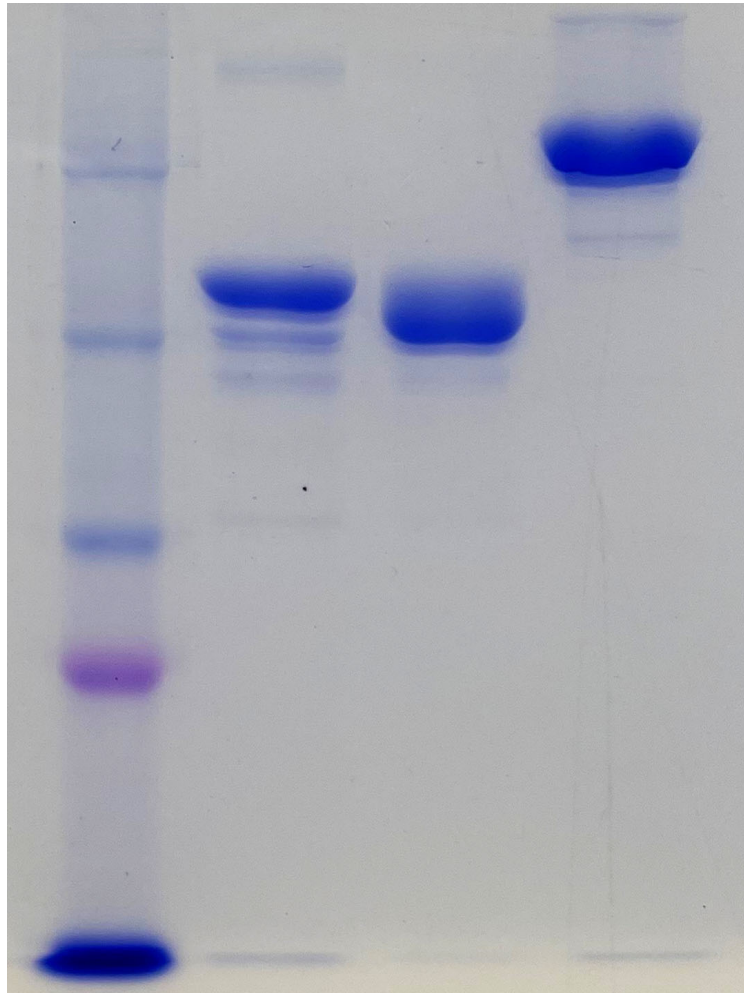

Figure 1B, part 2

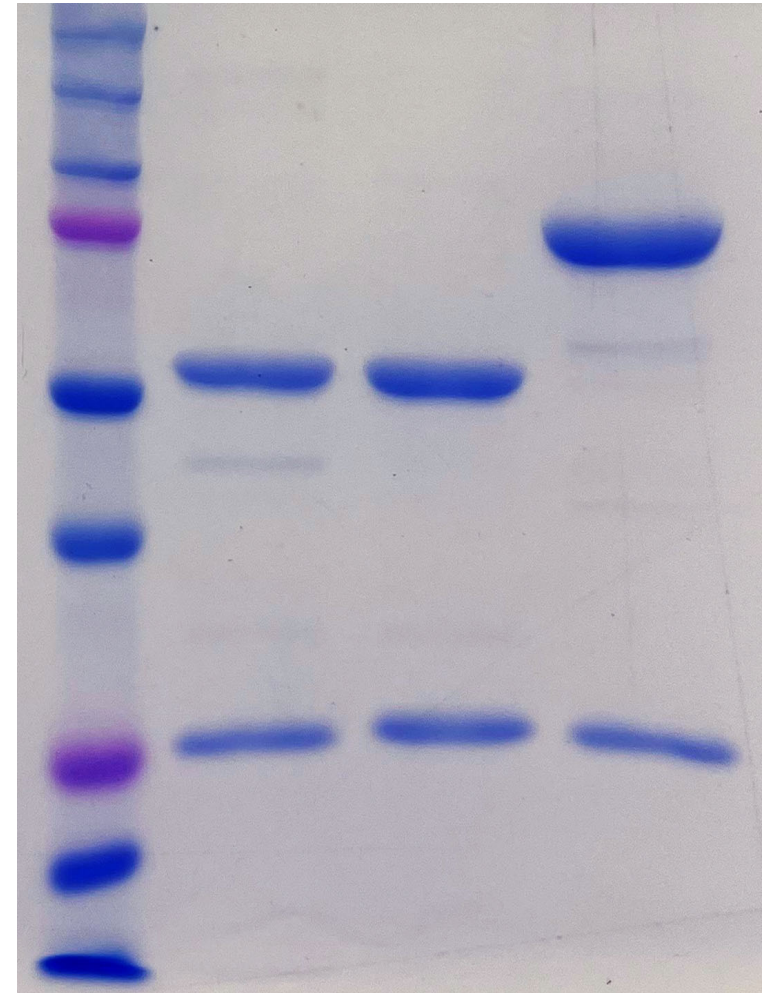

Supplement: Unedited blot and gel images [file jciinsight-10-194494-s177.pdf]
